# ImproWin® in the treatment of gastric ulceration of the squamous mucosa in trotting racehorses

**DOI:** 10.1186/1751-0147-56-13

**Published:** 2014-03-13

**Authors:** Ingunn R Hellings, Stig Larsen

**Affiliations:** 1 Rikstotoklinikken Bjerke AS, Postboks 194, Økern, N-0150 Oslo, Norway; 2 Department of Production Animal Clinical Sciences, Norwegian School of Veterinary Science, Postboks 8146 Dep, N-0033 Oslo, Norway

**Keywords:** Horses, Gastric Ulcer, ImproWin®

## Abstract

**Background:**

Gastric ulceration is highly prevalent in horses, and there is a large commercial market for feed-additives and non-licenced products that claim effect for prevention and treatment of gastric ulceration. ImproWin® has been used as a feed additive in horses with anecdotal evidence that it may have some positive effects on gastric ulceration.

The aim of this study was to investigate the effect of ImproWin® treatment on spontaneously occurring gastric ulcers of the squamous mucosa in Standardbred and Coldblooded trotting racehorses.

The study was performed as a randomised, double-blinded, single centre study with stratified semi cross-over design with breed as stratification factors. The horses were clinically and endoscopically examined prior to start and after three weeks of treatment. The ulcerations were scored in accordance with Equine Gastric Ulcer Council (EGUC) recommendations on a 5 point scale and on a 10 cm Visual Analogue Scale (VAS). The patients were responder-classified after 3 weeks. Responders in need of ulcer treatment were randomly allocated to 2 or 4 weeks of additional treatment. Non-responders to placebo were crossed to ImproWin®.

**Results:**

The 5-point EGUC score and VAS recorded score was significantly reduced (*P* ≤ 0.01) in both groups after 3 weeks of treatment. From 3 weeks to the end of treatment the score was further significantly reduced in the ImproWin® group (*P* ≤ 0.05).

At the end of treatment, 78% in the ImproWin® group and 54.8% in the placebo group were classified as responders. The difference was significant (*P* = 0.04).

**Conclusions:**

ImproWin® may aid the healing process of ulcers of the gastric squamous mucosa of trotters.

## Background

Gastric ulceration is frequent in all types of horses. Gastric ulcers were first recognised as an important disease of foals [1]. It has later been shown that gastric ulceration is highly prevalent in mature horses, especially those exposed to high levels of training and intensive management practices [2].

The prevalence of gastric ulcers reported in mature horses varies between populations and ranges from 58 to 93% [2-5]. In Standardbred trotters the prevalence has been found to be 44 to 70% [6,7] and the prevalence was significantly higher in active racing horses compared to those at rest.

The proton pump inhibitor omeprazole has been found safe and highly efficient in both treating and preventing recurrence of gastric ulcers in horses [8-11] at dose ranges from 1–4 mg/kg. Comparison of omeprazole and histamine 2 antagonists shows superior effect of omeprazole [12,13]. Other drugs used to enhance gastric mucosal protection, such as sucralfate and aluminium containing antacids, have shown inferior results or lack documentation of effectiveness in treatment of ulcerations of the equine squamous mucosa [14-16].

Calcium carbonate additives or alfalfa hay may reduce the ulcerogenic effect of volatile fatty acids from high starch diets and has been suggested as part of a preventive strategy. Pectin-lecithin complex has shown a possible positive effect on healing of naturally occurring gastric ulcers in horses [17,18]. However, the same product failed to prevent development of gastric ulcers in a group of feed deprived ponies [19]. Compounds containing antacids have shown variable effects and seem efficient in normalizing the pH only for a short period of time [20].

Methods to treat or prevent gastric ulcers effectively without requiring the continued administration of costly pharmaceutical agents and issues with withdrawal times would be highly desirable.

ImproWin®^a^ consists of salts of organic acids (SOC) in combination with B-vitamins and has been used in horses in Norway as a feed additive with anecdotal evidence that it may have some positive effects on gastric ulceration.

The aim of this study was to investigate the effect and benefit of ImproWin® treatment in spontaneously occurring gastric ulcers of the squamous mucosa in Standardbred and Coldblooded trotting racehorses.

## Methods

### Materials

The study population consists of Norwegian Coldblooded and Standardbred trotting racehorses past the age of 3 years having verified gastric ulceration of the squamous mucosa.

The Intention-To-Treat (ITT) material consists of 78 horses with endoscopically verified ulcers in the nonglandular mucosa of severity grade 2 or above on a scale from 0–4 [21]. Eleven horses, four in the ImproWin® group and seven in the placebo group, were blindly classified as violators to the protocol either due to use of other ulcer treatment or change in the feeding procedure during the study period. Of the remaining 67 horses included in the study 14 mares, 6 stallions and 16 geldings were allocated to ImproWin® and 12 mares, 5 stallions and 14 geldings to similar-looking same-volume placebo. The two treatment groups were found clinically equal regarding distribution of gender and breed. The mean age in the ImproWin® group was 5.3 years (range 2–8) and in the placebo group 5.8 years (range 3–13). No significant interaction was detected between the treatment groups, gender, breed and age (*P* > 0.21).

### Design and randomisation

The study was performed as a randomised, double-blinded and single centre study with stratified semi cross-over design [22]. Coldblooded and Standardbred trotters defined the study strata. Within stratum the horses were allocated 1:1 to ImproWin® or similar-looking same-volume placebo for three weeks by block randomisation with a fixed block size of eight [23]. Based on the improvement of 1 grade or more on the 5 point ulcer scale described below, the patients were classified as responder or non-responder to the given treatment. Patients classified as responder to the given three weeks treatment continued on the same treatment for an additional two or four weeks and endoscopically examined again. Patients in which the ulcers were grade 0 or 1 were considered cured and discontinued the study. Non-responders were blindly crossed to ImproWin® or proton pump inhibitors and treated conventionally in the clinic.

### Study procedure

All the included patients were recruited via Rikstotoklinikken Bjerke. Horses fulfilling the inclusion and exclusion criteria were included in the study when written consent from the owner and/or the trainer was given. Owners/trainers were asked to fill out a questionnaire recording any change in training/stabling/feeding between examinations.

The horses were clinically examined and the ulcer endoscopically verified and scored prior to start of treatment by one investigator (IRH). Prior to gastroscopy, food was withheld for 18–24 hours. Water was freely accessible up to the time of examination. A 3.3 meter videoendoscope^c^ was passed and the stomach manually insufflated with air through a garden spraypump system attached to the biopsy channel in the endoscope until the internal stomach folds appeared flattened. Feed material adherent to the non-glandular mucosa was flushed away with water through a separate garden spraypump system so the entire non-glandular portion of the stomach could be visualised, including the greater curvature, the lesser curvature and the dorsal fundus. The number and the degree of ulcers of the non-glandular mucosa were recorded in accordance with the Equine Gastric Ulcer Council (EGUC) recommendations [21]. The glandular area of the stomach was not evaluated for the purpose of this study. Additionally, the degree of ulceration was recorded on a 10 cm Visual Analogue Scale (VAS) by the investigator after each endoscopic examination. The VAS is a subjective overall score of the ulcer severity marked on a continuous scale from 0–10, where 0 is no ulceration and 10 is the maximum score. This scale is validated in humans [24] but not in horses. In accordance with the prerandomisation code, the treatment was started the same day as the initial examination. The treatment dose of ImproWin® and placebo^b^ was 40 grams (50 ml) powder once a day mixed in the feed with water. In the few cases where the horses refused to eat the powder mixed into the food, the powder was mixed with water into a paste and given in a syringe by mouth. The horses were all trained as normal during the treatment period and they were allowed to participate in races.

After three weeks of treatment a second endoscopic investigation was performed by the same endoscopist and the degree of ulceration was recorded in the same way as previously explained. Responders still in need of ulcer treatment were randomly allocated to either 2 or 4 weeks continuous treatment with the same regime and again endoscopically investigated. Patients with unchanged or increased degree of ulceration after three weeks of treatment were classified as non-responders to the given regime. Non-responders to placebo were crossed to ImproWin® treatment for either two or four weeks and finally endoscopically examined. The non-responders to ImproWin® were all crossed to proton pump inhibitor therapy and treated conventionally in the clinic, but not included in the study.

### Statistical analysis

Assumed continuously distributed variables are expressed by mean values with 95% confidence interval constructed by using the Student procedure [25]. Confidence intervals for prevalence were constructed using simple binomial sequences [26]. Discrete distributed variables are expressed in contingency tables [26]. All tests are performed two-tailed with a significance level of 5%. Analysis of Variance (ANOVA) with the initial degree of ulcer as covariate was performed for changes within and comparison between groups [27] on the assumed continuously distributed variable. Changes and comparison on discrete variables were performed by Contingency Table Analysis [26].

With a significance level of 5%, a power of 90% and a clinical relevant difference between the groups of one time the standard deviation, 32 horses in each group had to be included.

## Results

No significant difference (*P* = 0.33) was found at the start between the two treatment groups regarding degree of ulceration recorded on the equine gastric ulceration syndrome (EGUS) recommended scale. During the first three weeks of treatment, the ulcer score was reduced for 25 of the 36 (69.4%) ImproWin® treated horses and for 18 of the 31 (58.1%) in the placebo group. The reductions were found significant in both groups (*P* < 0.01).

At the start of treatment in the ImproWin® group, 10 horses had grade 2, 18 horses had grade 3 and 8 horses had grade 4 ulcerations (Table 1). After 3 weeks treatment, 11 horses were unchanged or worse, 12 were graded 0, five graded 1, ten graded 2, five graded 3 and four horses were graded 4. Consequently, an improvement of 1 grade was seen in eight horses, 2 grades in ten horses, 3 grades in three horses and 4 grades in four horses in the ImproWin® group. After 3 weeks treatment in the placebo group, 13 horses were unchanged or worse. An improvement of 1 grade was seen in five horses, 2 grades in ten horses and 3 grades in three horses. No horse had an improvement of 4 grades in the placebo treated group. No significant difference (*P* = 0.36) was detected between the groups on ulcer score after three weeks of treatment. From three weeks to end of treatment in the ImproWin® group the ulcer score was found to be reduced for 12 horses and increased in one. In the placebo group, six horses had a reduced score and two had an increased score during the same period. The reduction of ulcer score in the ImproWin® group was found significant (*P* < 0.01) but not in the placebo group (*P* = 0.15). The ulcer score was found significantly lower in the ImproWin® group compared to placebo group at the end of treatment (*P* ≤ 0.05).

**Table 1:** The degree and change in degree of ulceration from start to three week and to end of treatment

**Treatment**	**Weeks of recording**	**Degree of ulceration**	**Recorded degree of ulceration at start**			**Total**
			**2**	**3**	**4**	
ImproWin®	3 weeks	0	5	3	4	12
		1	1	4	0	5
		2	**3**	6	1	10
		3	0	**4**	1	5
		4	1	1	**2**	4
	Total		10	18	8	36
	End of treatment	0	6	10	6	22
		1	2	3	0	5
		2	**1**	1	0	2
		3	0	**3**	0	3
		4	1	1	**2**	4
	Total		10	18	8	36
Placebo	3 weeks	0	7	2	0	9
		1	3	0	1	4
		2	**2**	2	3	7
		3	2	**8**	0	10
		4	0	0	**1**	1
	Total		14	12	5	31
	End of treatment	0	10	4	1	15
		1	0	0	1	1
		2	**1**	0	1	2
		3	3	**8**	0	11
		4	0	0	**2**	2
	Total		14	12	5	31

The degree and change in degree of ulceration from start to three week and to end of treatment recorded on the 5 point scale [21]. The numbers bolded indicate the number of horses which were unchanged after the given treatment. The numbers above the bolded numbers indicate horses improved after the given treatment and the numbers below indicate the number of horses that had a worsening of ulcer score in that treatment period.

**Figure 1:**
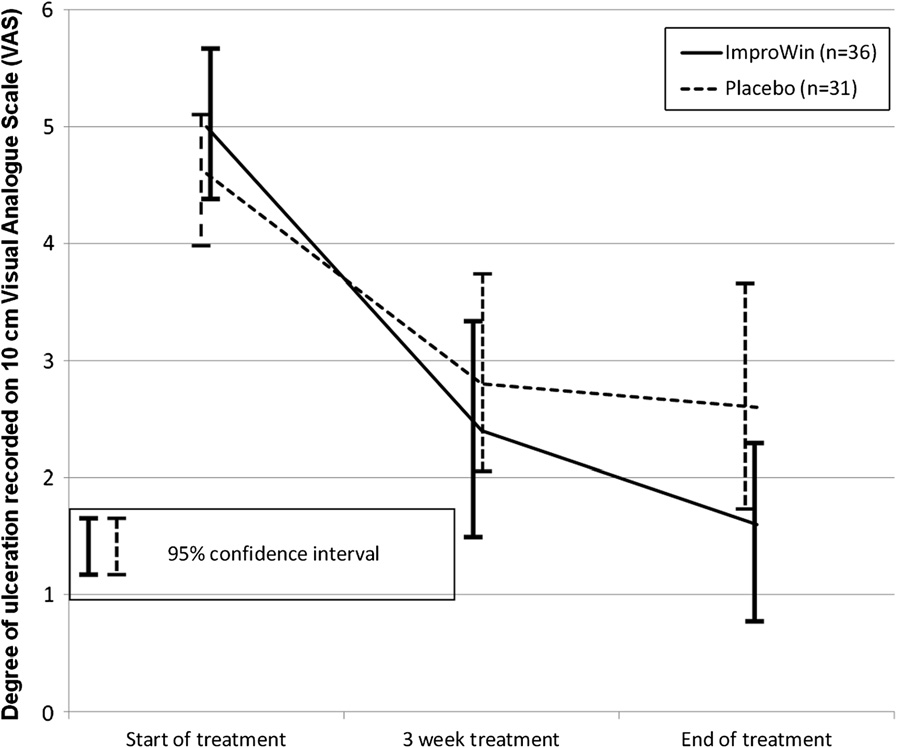
**Degree of ulceration recorded on 10 cm Visual Analogue Scale (VAS).** The degree of ulceration recorded on 10 cm VAS from start to three week and to end of treatment. The results are expressed as mean values with 95% confidence interval in brackets.

The two treatment groups were found comparable at start of treatment (*P* = 0.37) regarding the degree of ulceration recorded on a 10 cm VAS (Figure 1). In the ImproWin® group, the ulceration was significantly reduced (*P* < 0.01) from 5.0 (95% CI: 4.4 – 5.7) to 2.4 (95% CI:1.6 – 3 .3) after 3 weeks of treatment and further to 1.6 (95% CI: 0.7 – 2.6) at the end of treatment (*P* = 0.02). A significant reduction from 4.6 (95% CI: 4.0 – 5.2) to 2 .8 (95% CI: 1.9 – 3.7) was also detected in the placebo group (*P* < 0.01). A further reduction to 2.6 (95% CI: 1.7 – 3.5) at the end of treatment in the placebo group was not found significant. The total mean reduction of 3.4 (95% CI: 2.3 – 4.4) in the ImproWin® group was found significant larger than the comparable reduction of 2.0 (95% CI: 1.0 – 3.0) in the placebo group (*P* ≤ 0.05). The mean percent reduction in the VAS recorded degree of ulceration in the ImproWin® group was found to be 68.2% (95% CI: 49.3 – 87.1) and 36.1% (95% CI: 8.5 – 63.8) in the placebo group. The difference was found significant (*P* ≤ 0.05).

After three weeks of treatment, 69.4% of the horses in the ImproWin® group and 58.1% in placebo group were classified as responders to the given treatment (Table 2). The difference was not found significant (*P* = 0.48). Thirteen of the patients in the placebo group were classified as non-responders and 11 were crossed to four week ImproWin® treatment. Of these 11 horses, six were classified as responder to the active treatment. In the placebo group, 18 responders and two non-responders continued the same treatment and 17 of these were classified as responders at the end of treatment. At the end of treatment, 54.8% of the horses in the placebo group and 78% in the ImproWin® group were classified as responders. The difference was significant (*P* = 0.04).

**Table 2:** Classification of responders after 3 weeks and at the end of treatment

**Treatment period**	**a) ImproWin®/ImproWin® (n = 36)**	**b) Placebo/ImproWin® (n = 11)**	**c) Placebo/Placebo (n = 31/n = 20)**
3 weeks	25/36	0/11	18/31
	69.4 (51.9 – 83 .7)	0.0 (0.0 – 28.5)	58.1 (40.0 - 74.1)
End of treatment	28/36	6/11	17/31
	77.8 (60.9 – 89–9)	54.6 (23.4 – 83.3)	54.8 (34.7 – 73.0)

Classification of responders after 3 weeks and at the end of treatment in the groups given a) Only ImproWin® b) Placebo and ImproWin® the last part and c) Only placebo. The results are expressed as number of responders and percent responders with 95% confidence interval.

## Discussion

The significant improvement in degree of ulceration score in the placebo group (58.1%) found after 3 weeks of treatment was higher than reported in previous studies. Spontaneous improvement rates of 32% compared to 99% improvement in horses treated with omeprazole were found after 28 days in a similar study. The overall healing rate was 8.9% and 87% respectively in the control and omeprazole treated groups in the same study [11]. In that study the personnel that administered the medication was not able to observe the examination. Another study evaluating omeprazole in the treatment and prevention of gastric ulceration in racehorses demonstrated an improvement in ulcer score of 48% after 13 days, 42% after 20 days and 32% after 27 days in the control group [9]. In this study all the horses were reported to be treated equally regarding feed and management. The healing rates in the control groups were 4, 8 and 4% respectively. Treatment with omeprazole at 4 mg/kg resulted in 78% healing rates after 28 days, while 92% were significantly improved.

Environmental factors such as feed, training/exercise and housing are well known to influence development and healing of gastric ulcers in horses. All owners or trainers were told the results of the gastroscopy in our study. Although they were instructed not to perform any differences in feeding or training regimes, it is reasonable to assume that a significant proportion did instigate changes that would very likely influence the development and healing of the ulcers. Due to the blinding of the treatment, these changes have to be assumed equal in the two groups. It was decided to keep these variables uncontrolled due to the difficulty in controlling exactly what the owners and trainers were feeding the horses.

Despite the lack of significant difference in prevalence of responders between the two treatment groups after 3 weeks, there was a significant different at end of treatment in favor of the ImproWin® group. This difference is based on the assumption that horses not responding to treatment after 3 weeks would still be non-responders at the end of treatment. All non-responders to placebo were crossed to ImproWin® with more than 50% response to treatment. On the contrary, the three horses that did not respond to ImproWin® were classified as responders at the end of treatment, even though these 3 horses should have been crossed to proton pump inhibitors. This would have given an advantage for the ImproWin® group end results.

The non-responders to placebo after 3 weeks were switched to ImproWin® for either 2 or 4 weeks. The obtained response rate was lower than the initial response rate in the ImproWin® group after 3 week.

The length of ImproWin® treatment seems to have an impact on the response rate, with lowest prevalence after 2 and highest after 7 weeks of treatment. The improvement of 4 grades was seen in four horses after 3 weeks and six horses at the end of treatment in the ImproWin® group. Comparatively, an improvement of 4 grades was not seen in any of the placebo treated horses after 3 weeks and in only one horse at the end of treatment.

A few trainers/owners in our study reported that the feeding and environment were changed significantly and these were classified as violators and excluded from the study. The classification of violators was performed blindly and it is believed likely that change in management and feeding would be equally reflected in both treatment groups. The study was performed double-blinded, but it is still possible that the trainers and owners were able to identify the treatment given due to some differences in smell and color. This could also influence the obtained results. Many studies focus on the incidence of EGUS in horses under different management, exercise and feeding regimes [20,28-34]. Part of the spontaneous improvement may be due environmental changes and factors and natural healing. We were unable to separate these factors in our study since there was no attempt to standardise management and feeding protocols.

Salts of organic acids (SOC) have been used as a growth promoter in pigs and research in this species has found that SOC influence the bacteria in the proximal gastrointestinal tract *in vitro*[35,36] without negative effect on the stomach lining [37]. The potential acidifying effect of the use of SOC on horses’ gastric pH would obviously be a concern when using these products to treat or prevent EGUS. Although it is generally considered that dietary organic acids or their salts lower gastric pH, this has been difficult to demonstrate [38]. When SOC are added into an acidic environment, the solution will still be acidic but pH will be slightly higher [39] and these compounds may therefore act as a buffer of strongly acidic solutions such as in the stomach. It is possible that the adding of an organic acid reduce the parietal acid secretion in the stomach although the precise mechanism is not known.

The influence of these SOC-containing products on the stomach pH has not been measured in horses, but measurements in the caecum after feeding SOC to pigs showed increase in pH compared to the pH in control pigs [40]. In addition, several investigations have shown that these products have a strong bactericidal effect without significantly decreasing the pH values in the gastrointestinal tract [35].

ImproWin® is well documented to inhibit pathogenic bacteria *in vitro*, including *Helicobacter* and other bacteria such as *Escherichia coli*[36]. *Helicobacter*-like DNA was found in the gastric mucosa of horses with gastric ulcerations [41], but so far there have been no conclusive findings of a possible pathogenic link between presence of *Helicobacter*-like DNA and clinical manifestation of EGUS. Bacterial colonization by Gram negative bacteria in established gastric ulcerations in rats has been shown to delay healing while *Lactobacillus* colonization had a protective effect in the same study [42]. Bacteria have also been found to exacerbate mucosal injury in ulceration in the stomach or small intestine induced by non-steroid anti-inflammatory drugs [43]. Organic acids are believed to enter the microbial organism in the undissociated form and dissociate in the more alkaline cell interior, causing acidification of the cytoplasm and inhibition of metabolism. This effect is more pronounced in the acid environment of the stomach compared to the less acidic small intestine environment [36]. Bacteria, including *E. coli*, were cultured from equine stomachs [3] and it is possible that ImproWin® has a beneficial effect on healing of gastric ulcers by reducing the negative effect of bacterial colonization.

Another possible harmful effect caused by the presence of pathogenic bacteria in the stomach is production of volatile fatty acids (VFAs) and lactic acids from fermentation of soluble carbohydrates. VFAs and HCl have been shown to penetrate the gastric mucosa at low pH and disrupt cellular transport and cause cell swelling, cell death and ulceration *in vitro*[44]. However, the bactericidal effect of ImproWin® in EGUS in horses remains unclear.

**Figure 2:**
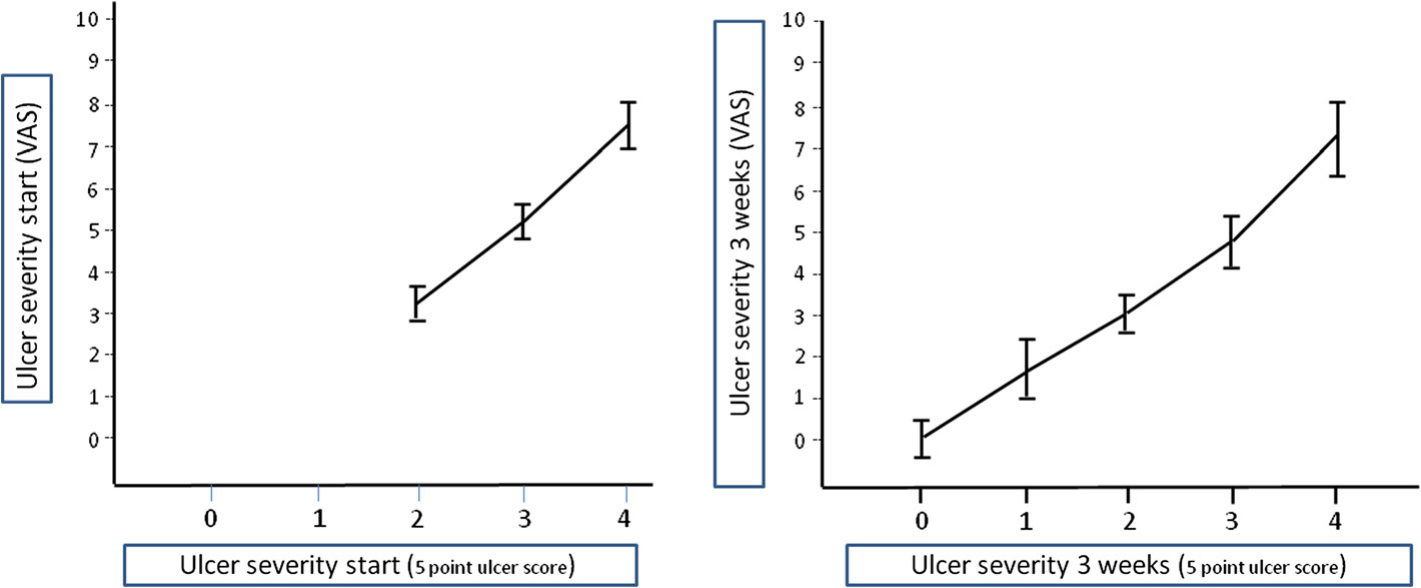
**Agreement on ulcer severity between Visual Analogue Scale (VAS) and 5 point scale.** Ulcer severity recorded on 5 point scale [21] and 10 cm VAS at the start and after 3 weeks of treatment. The results recorded on the VAS are expressed as mean values with 95% confidence intervals within each ulcer grade.

The present study shows a significant reduction in ulcer score after 3 weeks of treatment in both groups. The reduction in degree of ulceration from start to end of treatment was found significantly larger in the ImproWin® group compared to the placebo group, both with the 5 point scale and VAS. Comparison of the results obtained by using the 5 point EGUC recommended scale and the VAS detected a surprisingly linear relation (Figure 2). This is probably because the scoring was always performed by the same operator, with several operators the deviation would increase. Assuming these important limitations, it appears that VAS can give important additional information outside the 5 point scale when using only one operator. With a five point fixed scale a large number of patients would be needed to discover a difference. By using VAS we were able to detect smaller differences with a limited number of patients. Based on clinical trials in human, the VAS in evaluation of mucosal lesions is recommended [24] but has not been validated in horses. To evaluate this scoring system one would need to design a separate study using more than one evaluator. It may be more appropriate to use as a VAS scale for grading glandular ulcerations (as in humans) where the ulcers tend to be less variable in appearance.

The use of ImproWin® has not been validated against the use of proton pump inhibitors. However, issues with withdrawal times and cost of treatment with omeprazole are of practical concern when treating ulcers in the non-glanduar mucosa in racehorses. It is the opinion of the authors that ImproWin® may have a place in treating and possibly preventing these ulcers in horses while racing since the supplement is allowed to use while racing in Norway at the time of writing. Our results suggest that treatment rates improve with longer periods of treatment, however, response rate according to weeks of treatment was not analysed separately in this study because there were too few horses in each group and further studies would be necessary to evaluate this.

## Conclusion

ImproWin® seems to have a positive effect on ulcer healing and adds benefit to the healing of gastric ulcers in the squamous mucosa in horses on top of management changes and placebo effect.

## End notes

^a^ImproWin®: HCOONa (Sodium formate), Ca(HCOO)_2_ (Calcium formate), C_4_H_2_FeO_4_ (Iron fumarate), C_19_H_19_N_7_O (Folic acid), C_63_H_88_CoN_14_O_14_P ( Cobalamin), C_8_H_11_NO_3_ (Pyridoxine). Approx. pH of dry powder: 10. Vitality Innovation, Oslo, Norway.

^b^Placebo: icing sugar, paprikapowder (tasteless/tastepoor) and turmeric.

^c^Videomed, Targetstr 2, Munchen, Germany.

## Competing interests

The project has been partly funded by Vitality Innovation AS which is the company that own and has patent on ImproWin1. Vitality Innovation is paying for the article processing charge. The authors have no personal financial or non-financial competing interest in the product.

## Authors’ contribution

All authors have contributed significantly and are in agreement of the content of the manuscript. IRH has performed the gastroscopies and have drafted the main parts of the article under guidance of SL. SL has designed the study, performed the statistical analysis and written the sections on the materials and results and made the tables and figures. Both authors have read and approved the final manuscript.
